# SARS-CoV-2 antibody–dependent enhancement of infection depends on antibody binding to both ACE2 and Fc receptors

**DOI:** 10.1172/jci.insight.197773

**Published:** 2026-02-23

**Authors:** Natalia A. Kuzmina, Sivakumar Periasamy, Kritika Kedarinath, Keziah Hernandez, Caroline Atyeo, S. Moses Dennison, Kan Li, Daniel Bedinger, Sharon L. Schendel, Georgia D. Tomaras, Hanif Ali, Galit Alter, Erica Ollmann Saphire, Alexander Bukreyev

**Affiliations:** 1 Department of Pathology, University of Texas Medical Branch (UTMB), Galveston, Texas, USA.; 2 Galveston National Laboratory, Galveston, Texas, USA.; 3 Ragon Institute of MGH, MIT, and Harvard, Cambridge, Massachusetts, USA.; 4 Center for Human Systems Immunology, Departments of Surgery, Immunology, and Molecular Genetics and Microbiology and Duke Human Vaccine Institute, Duke University, Durham, North Carolina, USA.; 5 Carterra Inc. Salt Lake City, Utah, USA.; 6 Center for Infectious Disease and Vaccine Research, La Jolla Institute for Immunology, La Jolla, California, USA.; 7 Quadrucept Bio Ltd, Kemp House, London, United Kingdom.; 8 Department of Medicine, University of California San Diego, La Jolla, California, USA.; 9 Department of Microbiology and Immunology, University of Texas Medical Branch, Galveston, Texas, USA.; 10 Center for Biodefense and Emerging Viral Infections, University of Texas Medical Branch, Galveston, Texas, USA.

**Keywords:** Immunology, Virology, Adaptive immunity, COVID-19, Immunoglobulins

## Abstract

Antibody-dependent enhancement (ADE) of infection is a well-described phenomenon for several viruses, including dengue, Ebola, respiratory syncytial virus, and HIV. ADE occurs when virus-antibody complexes engage Fc receptors (FcRs) and virus-specific receptors, enhancing infection under conditions of incomplete neutralization. The Coronavirus Immunotherapeutic Consortium (CoVIC) assembled a comprehensive dataset of functional properties for over 400 mAbs, enabling direct comparison of neutralization, Fc-mediated functions, receptor binding, and infection of immune cells. Infection rates in most primary human immune cell types were low, with modest increases observed for some mAbs. In contrast, macrophages were more susceptible to SARS-CoV-2 and exhibited substantial ADE with select mAbs. ADE was completely inhibited by FcR blockade and significantly reduced by antibody- or ceftazidime-mediated blocking of angiotensin-converting enzyme 2 (ACE2). Neutralization potency did not correlate with ADE, as both strongly and weakly neutralizing antibodies induced enhancement. Instead, ADE magnitude depended on an antibody’s ability to block spike protein binding to ACE2. Importantly, ADE resulted in productive infection with release of infectious virus. Evaluation of antibodies against the BA.1 (Omicron) variant revealed reduced or lost ADE for most mAbs, with increased ADE observed for several mAbs relative to the USA-WA1/2020 strain.

## Abstract 2

Some of antibodies induced by SARS-CoV-2 facilitate enhancement of infection which depends on antibody binding to both ACE2 molecule and Fc receptors on immune cells.

## Introduction

The Coronavirus Immunotherapeutic Consortium (CoVIC) comprising 56 partners across the world was created for global partnership to accelerate discovery, optimization, and delivery of life-saving antibody-based therapeutics against SARS-CoV-2. The CoVIC alliance managed contribution of multiple antibodies and comparative investigation of their binding to the target, neutralizing activities, binding to Fc receptors (FcRs), Fc-mediated effects, epitopes, and protective efficacy in animal models (1), resulting in the creation of a database (2) allowing a direct comparison of a large number of mAbs.

Antibody dependent enhancement (ADE) of infection is a well-known phenomenon described for multiple viruses: dengue (3, 4), respiratory syncytial virus (5), influenza (6, 7), and Ebola (8–10). As shown for many viral infections, antibodies may enable viral entry into FcγR-bearing cells, bypassing specific receptor-mediated entry, and can amplify the number of virions entering cells or enhance infection by involving immune mechanisms (11). If these antibodies are unable to neutralize viruses, this increased entry leads to an enhancement of a productive infection (11). ADE was documented for several coronaviruses as well: Middle East respiratory syndrome–related coronavirus (MERS) (12), SARS-CoV (13–16), and feline infectious peritonitis virus (FIPV) (17–20), but it never appeared to be as strong as was described for dengue virus. Dengue differs from other viruses because it targets monocytes, macrophages, and DCs and can produce progeny virus in these cells (4). Unlike dengue, the preexisting immunity gained from previous exposure with other coronaviruses does not promote ADE during infection with SARS-CoV-2 (21), suggesting that SARS-CoV-2, SARS-CoV, and MERS-CoV have a significant antigenic distance, and the observed cross-reactivity with SARS-CoV and MERS-CoV spike protein (22) does not contribute to the enhancement. SARS-CoV-2 uses angiotensin-converting enzyme 2 (ACE2) as a receptor to infect host cells (23, 24). ACE2 expression in primary immune cells varies broadly; macrophages demonstrate detectable levels of ACE2 (25–27). Compared with primary macrophages, THP-1 cells show lower baseline ACE2 expression, but their response to stimuli mirrors primary cells (28–30). After recognition of ACE2 by the spike protein on the surface of SARS-CoV-2 and membrane fusion, virus enters target cells (24, 31). Though SARS-CoV uses the ACE-2 receptor for entry as well, the binding affinity of SARS-CoV-2 to this receptor is up to 20-fold higher compared with SARS-CoV, making it pivotal for higher infectivity of SARS-CoV-2 compared with other coronaviruses (32). The receptor binding domain (RBD) of the spike protein is a key target for neutralizing antibodies (33) and is highly variable between individual coronaviruses; in addition, its mutations allow the virus to evade the antibody response (34). RBD plays a central role in this entry. Blocking of binding of the spike protein to ACE2 is an effective way to inhibit the infection of target cells by SARS-CoV-2 (35, 36). 

For SARS-CoV-2, at least 2 case reports described ADE that possibly occurred due to the infusion of mAbs and led to an acute boost in the severity of COVID-19 pneumonia (37, 38). Importantly, 2 mAbs used for treatment of SARS-CoV-2, casirivimab and imdevimab, were found to induce ADE in vitro (39). Recent reports have demonstrated that some plasma samples from patients with COVID-19 can enhance SARS-CoV-2 infection only in cells expressing both FcR and ACE2 (40, 41), and SARS-CoV-2 may promote ACE2 expression in immune cells (42–45). Moreover, ACE2 may act as a secondary receptor required for antibody- and FcγR-mediated elevated entry of SARS-CoV-2 (46). This study aimed to investigate the relative contributions of FcγRs, ACE2, neutralization potencies, and epitope specificity in ADE. We used a large and diverse panel of antibodies to understand how these factors influence ADE and to identify the most significant factors contributing to ADE. We show that ADE caused by these antibodies can be inhibited by blocking either the FcγR or ACE-2, suggesting a dual mechanism of the enhancement.

## Results

### SARS-CoV-2 ADE is mediated by both neutralizing and non-neutralizing antibodies in a dose-dependent manner.

**Figure 1:**
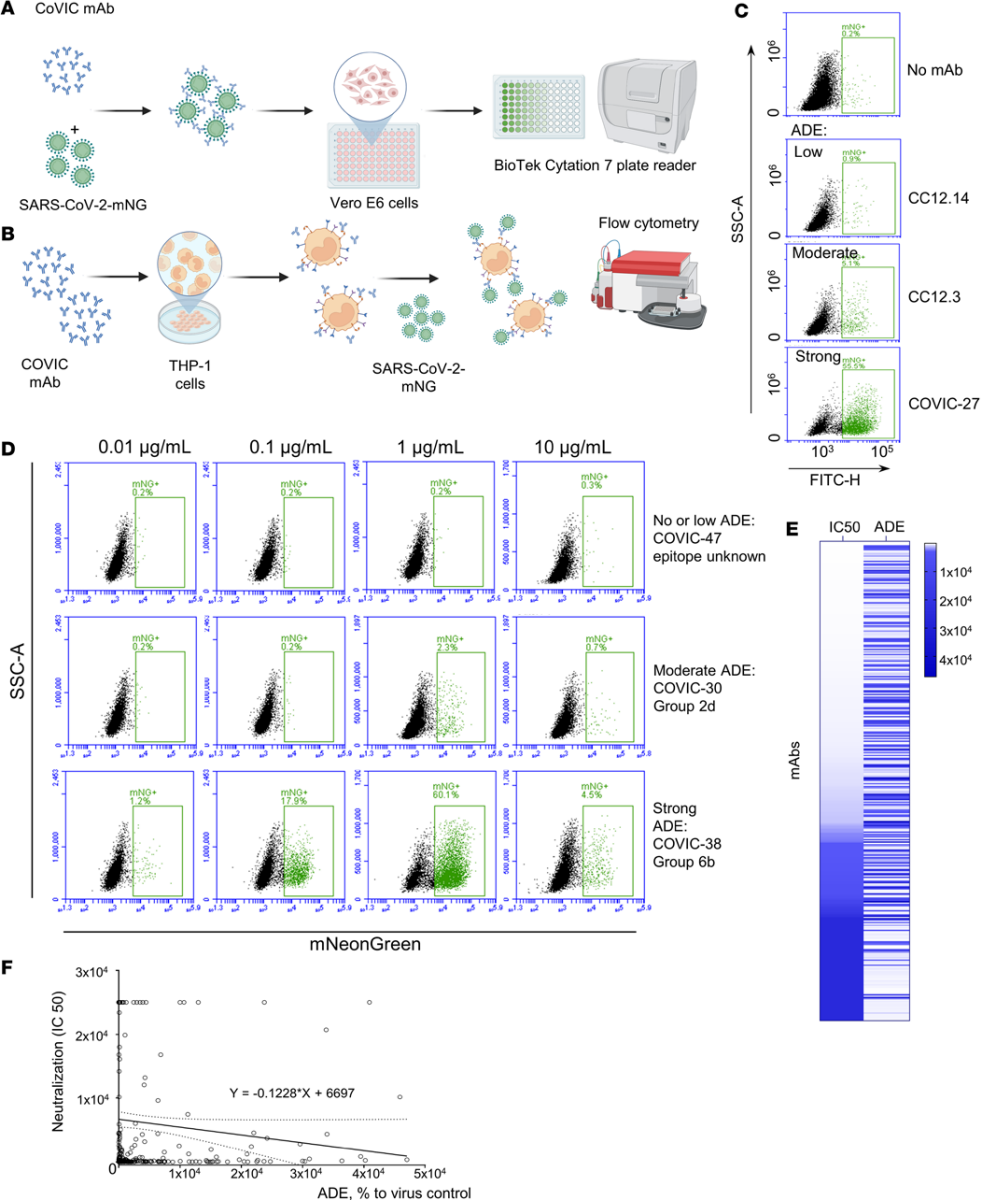
Both neutralizing and non-neutralizing mAbs increase susceptibility of THP-1 cells to SARS-CoV-2. (**A**) Schematic of virus neuralization assay. (**B**) Schematic of assessment of ADE. (**C**) Various mAbs (indicated at right) demonstrate different extent of ADE (tested at concentration of 1 μg/mL): no or low (<2% infected cells); moderate (2%–20% infected cells), and strong (>20% infected cells). CC12.3 and CC12.14 served as control antibodies for the CoVIC panel (1). (**D**) ADE at various mAb concentrations in THP-1 cells: no or low ADE, moderate ADE, strong ADE. For most of the mAbs, the greatest enhancement was observed at 1 μg/mL. The antibodies are indicated at the right. (**E**) Heatmap of tested mAbs ranked by IC_50_ and the extent of ADE. (**F**) Absence of a direct correlation between neutralization and ADE, *R*^2^ = 0.0097 for simple linear regression, IC_50_ in ng/mL and ADE mediated by antibodies used at the dose 1 mg/mL, percentage of infection with no mAb control.

CoVIC has analyzed a panel of 407 mAbs in multiple in vitro and in vivo assays and comparative data has been uploaded into a publicly accessible database (https://covic.lji.org). To monitor viral infection, we used a recombinant SARS-CoV-2 expressing neon green (SARS-CoV-2-mNG), which allows quantification of virus-infected cells based on fluorescence of neon green intracellularly produced from virus-encoded mRNA (47). We measured neutralizing potencies and ADE at various concentrations of mAbs in vitro (Figure 1A) and analyzed their properties, available from the database, which can affect their abilities to decrease or increase viral infection. For this work, 364 IgG1 antibodies from the CoVIC panel were selected, the majority of which were nonmodified mAbs. For comparative purposes, we also included several engineered multivalent antibodies (Supplemental Figure 1; supplemental material available online with this article; https://doi.org/10.1172/jci.insight.197773DS1). Their binding magnitude to various FcRs were comparable with the entire analyzed dataset (Supplemental Figure 2), which is shown in https://covicdb.lji.org/ The monocytic cell line THP-1 was treated with a panel of 364 selected mAbs at concentrations of 10, 1, or 0.1 μg/mL, and SARS-CoV-2-mNG was added at MOI 3 PFU per cell. Cells were incubated for 48 hours and the numbers of infected (mNG+) cells were quantified by flow cytometry (Figure 1, B–D). We observed dose-dependent enhancement of infection (ADE), with a maximum at concentration of 1 μg/mL for the most mAbs, and a typical dependence of ADE magnitude on mAb concentration: the increase of ADE as concentration decreased from 10 μg/mL to 1 μg/mL, and then ADE subsequently decreased at lower concentrations. In contrast, no ADE was observed with the VIC16 mAb specific for Ebola virus (48) (Supplemental Figure 3). In a separate experiment, the viability of infected cells treated with representative SARS-CoV-2 mAbs or an irrelevant control antibody was confirmed (Supplemental Figure 4). Furthermore, we demonstrated that 2 representative mAbs that caused strong ADE (CoVIC-27 and CoVIC-113), 2 representative mAbs that caused no ADE (CoVIC-41 and CoVIC-147), and an irrelevant antibody bound FcRs on THP-1 cells with a similar efficiency (Supplemental Figure 5). These data suggest that ADE occurs under conditions of incomplete virus neutralization. Because of the drop of enhancement at the lowest concentration evaluated — 0.01 μg/mL in preliminary experiments — this concentration was excluded from further evaluation. The mean increases compared with no antibody control based on all mAbs tested were 429%, 3,814%, and 2,319% at 10, 1, and 0.1 μg/mL, respectively (*P* = 0.0001, 1-way ANOVA). The percentages of infected cells or the extent of ADE caused by mAbs at 1 μg/mL were considered the most representative and used for comparison of mAbs. Unexpectedly, we observed a lack of correlation between the neutralization potencies of mAbs (IC_50_) and their ability to mediate ADE (percentage enhancement compared with no antibody control), *R*^2^ = 0.0098. Moreover, some very potent antibodies caused strong ADE (Figure 1, E and F).

### SARS-CoV-2 ADE is preferentially mediated by antibodies that inhibit spike protein binding to ACE2 receptors.

**Figure 2:**
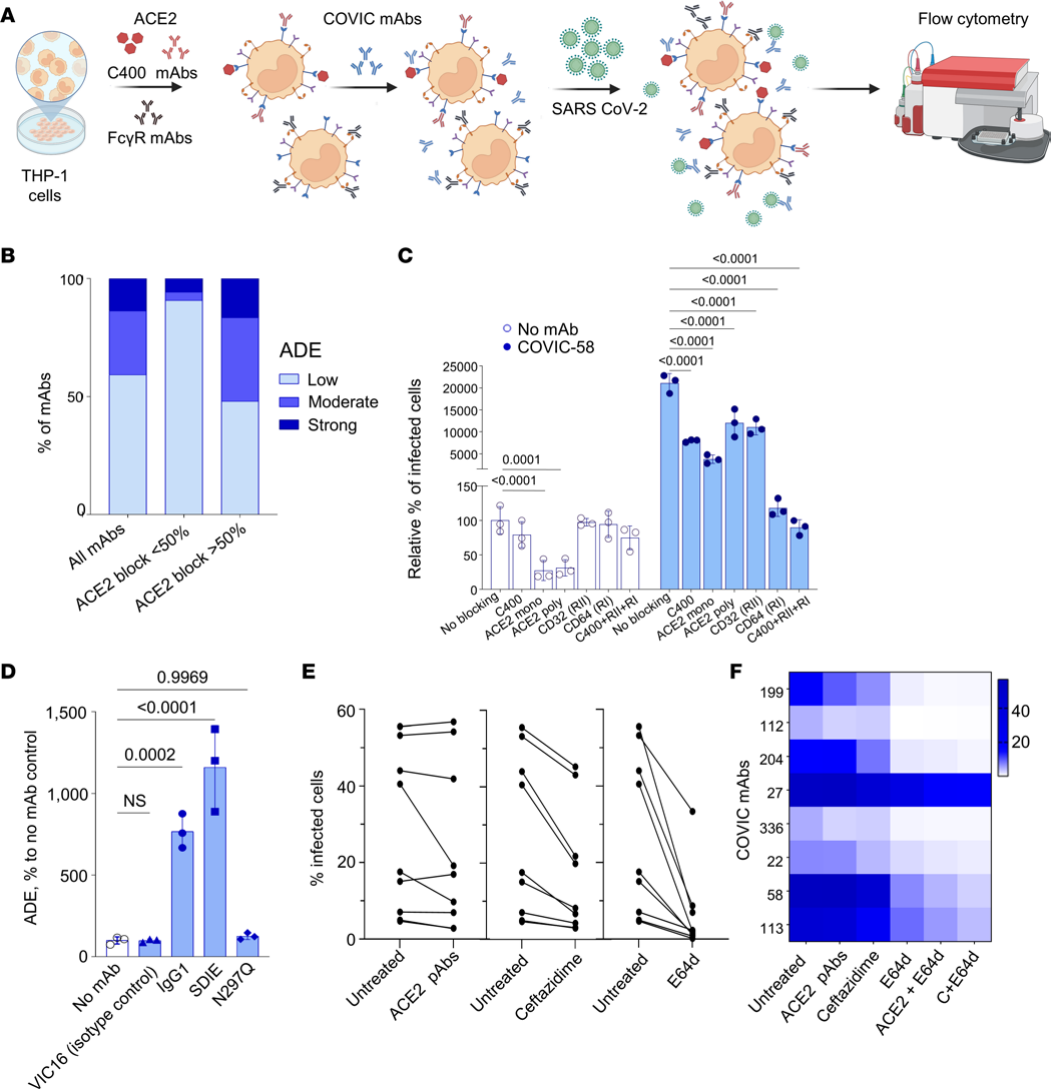
The extent of ADE depends on binding to ACE2 and Fcγ receptors. (**A**) Schematic of the experiments. (**B**) Proportion of mAbs causing low, moderate, and strong ADE differs among mAbs that block viral binding to ACE2 weakly versus strongly. (**C**) Blocking of ACE2 receptors with indicated polyclonal antibodies (10 μg/mL), chemical inhibitors ceftazidime (400 μM), dalbavancin (25 μM), or FcγRI or FcγRIIa receptors with anti CD32 or CD64 antibodies significantly reduces ADE mediated by CoVIC-58. Relative percentages of infected cells normalized to no antibody control. Tukey’s multiple-comparison test. (**D**) Effects of FcR modifications on ADE tested with the CR3022 mAb. VIC16, which is an IgG1 specific for Ebola virus glycoprotein (48), included as isotype control. Dunnett’s multiple-comparison test. NS, not significant. (**E**) Effects of blocking of ACE2 receptors and lysosomal cathepsin inhibitor E64d on ADE. (**F**) Heatmap of the relative effects of each treatment on ADE caused by individual mAbs assessed by percentage of infected cells. Treatment with ceftazidime or anti-ACE2 polyclonal antibodies and endosomal inhibitors has a cumulative effect, suggesting distinct mechanism of ADE inhibition.

We distinguished 2 sets of antibodies by their effectiveness in obstructing viral binding to ACE2 receptors: those with low (<50%) and with high (>50%) blocking capabilities, based on the inhibition measured by biolayer interferometry (49). The proportions of antibodies mediating low ADE (<10-fold compared with no mAb control or 2% infected cells), moderate ADE (10–100-fold, or 2%–20% infected cells), or high ADE (>100-fold, or >20% infected cells) varied across these 2 groups (Figure 2, A and B). Antibodies that were less effective in blocking binding of RBD to ACE2 demonstrated low ADE in most cases. In contrast, many of the antibodies that significantly reduced binding of RBD to ACE2 were associated with strong ADE. This difference between the antibody groups was significant (χ^2^ test, *P* < 0.0001), indicating that the ability of antibodies to block spike binding to ACE2 receptors is implicated in ADE.

### The magnitude of ADE depends on binding to ACE2 and Fcγ receptors.

To investigate the involvement of ACE2, which is the receptor of SARS-CoV-2 in ADE mediated by the virus, we utilized THP-1 cells (14, 50). The THP-1 cells used in our experiments demonstrated detectable expression of ACE2 (Supplemental Figure 6). We blocked binding of the antibodies to ACE2 by either ceftazidime, which directly interacts with the S-RBD (35) at 400 μM, or by mono- and polyclonal ACE2 blocking antibodies at 10 μg/mL for 1 hour. The cells were inoculated with SARS-CoV-2 at MOI 3 PFU per cell and incubated for 24 hours. The optimal concentration of ceftazidime was identified in preliminary experiments. We observed a significant decrease of infection when ACE2 receptors were blocked with ceftazidime or with mono- and polyclonal antibodies against ACE2 (Figure 2C). Next, we incubated THP-1 cells with anti-CD32 (FcγIIa) and anti-CD64 (FcγI) mAbs for 1 hour to block FcγRs, added CoVIC-58 mAb at 1 μg/mL, inoculated the cells with SARS-CoV-2 at MOI 3 PFU per cell, and incubated the cells for 24 hours. Blocking of FcγRIIa or FcγRI reduced ADE; in the latter case, the effect was more pronounced, which can be explained by a higher affinity of FcγRI compared with FcγRIIa (51). The partial inhibition of ADE by FcγR-blocking antibodies alone suggests that FcγRs are not the sole mediators of ADE. The complete abrogation of ADE observed with the combined use of ceftazidime and anti-FcγR antibodies supports the involvement of ACE2 in the enhancement mechanism (Figure 2C).

To further investigate the significance of FcRs in ADE, we evaluated antibodies with mutations within their Fc domain: N297Q, which completely eliminates the antibody’s ability to interact with the FcRs (52) and S239D/I332E (SDIE), which enhances Fc functions (53) (Figure 2D). We selected the CR3022 mAb, which is specific for SARS-CoV and capable of binding to but not neutralizing SARS-CoV-2. At 1 μg/mL, CR3022 in the form of IgG1 caused low ADE, which was completely abrogated by the mutation N297Q but significantly increased when the antibody version with the SDIE mutation was used. Importantly, in our previous studies, we demonstrated that this Fc modification exacerbated the severity of COVID-19 in antibody-treated mice, which was evidenced by increased viral load in the lungs and a greater loss of weight (54).

Cathepsin L plays a key role in SARS-CoV-2 infection in humans and is required for virus cell entry (55). To test whether the cysteine protease cathepsin L affects ADE, we administered the E64d inhibitor to THP-1 cells (Figure 2E). We observed a considerable decrease in the infected cell count across all tested enhancing mAbs, suggesting that cathepsin cleavage is required for effective ADE. The concurrent treatment with ACE2 and E64d inhibitors demonstrated a cumulative effect, indicating that these inhibitors suppress virus entry through distinct mechanisms (Figure 2F).

### Epitope specificity has only a limited effect on the magnitude of ADE.

**Figure 3:**
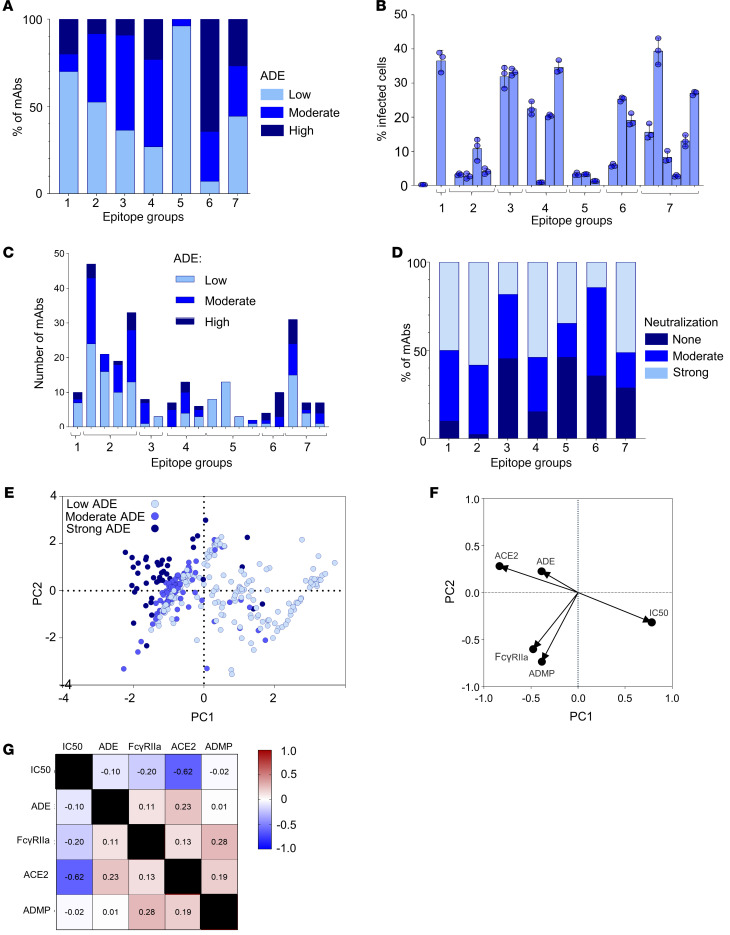
The extent of ADE depends on binding to ACE2 and Fcγ receptors and less on epitope groups. (**A**) Proportion of mAbs from different epitope groups mediating low, moderate, and high ADE based on percentage relative to no mAb control. (**B**) Percentages of infected THP-1 cells treated with the selected mAbs of various epitope groups (shown under the graphs) and subgroups: 1, 2a, 2b, 2c, 2d, 3a, 3b, 4a, 4b, 4c, 5a, 5b, 5c, 5d, 6a, 6b, 7a, 7b, and 7c at 1 μg/mL. (**C**) Proportions of mAbs mediating low, moderate, and high ADE within epitope groups and subgroups described for **B**. (**D**) Proportion of mAbs from different epitope groups that cause no, moderate, or strong neutralization based on IC_50_. (**E**) Multivariable analysis reveals distinct clusters of mAbs with low, moderate, or strong ADE based on their ability to block ACE2 receptors, the FcγRIIa activity, neutralization IC_50_, ADE, and ADMP. (**F**) A loadings plot showing a relationship between ADE and ACE2, and between ADMP and FcγR activities. (**G**) Spearman’s correlation matrix of variables used for the principal component analysis.

In our previous study, antibodies that bind to soluble RBD were grouped into 7 primary epitope groups, known as RBD-1 through RBD-7. These groups were further divided into subgroups according to the level of pairwise competition with other subgroups (1, 56). A set of mAbs, each specific to 1 of the 7 epitope groups, was chosen and evaluated for their capacity to facilitate ADE (Figure 3A). Antibodies targeting epitope groups 5 and 6 exhibited markedly different ADE profiles. Most mAbs from group 5 showed minimal ADE, despite their interaction with the RBD’s outer surface (1). Conversely, mAbs that target epitope group 6, which attach to the RBD’s inner surface, displayed high ADE (Figure 3A). These epitope groups also presented a consistent pattern at the subgroup level in their capacity to enhance ADE (Figure 3, B and C). However, for other epitope groups, no clear link was found between the specific groups and the extent of ADE, suggesting that the exact location of the binding site may be not a major factor in ADE. Next, we tested the neutralizing activities of the mAbs (Figure 3D), which suggested that the pronounced ADE observed in mAbs belonging to epitope group 6 could be due to their reduced neutralizing effectiveness. Although the majority of group 5 mAbs, which weakly block binding of RBD to ACE2, were also found to have low neutralizing activity, those that effectively block binding of RBD to ACE2 exhibited potent neutralizing capabilities. Remarkably, mAbs belonging to subgroups 5a and 5b, which have low neutralizing activity, also have a low affinity to the spike protein; these mAbs nonetheless demonstrate good protection in vivo (56). These antibodies are able to cross-link adjacent spikes together (1), and their enhanced in vivo protection despite moderate in vitro neutralization may be linked to this cross-linking ability.

Out of the 329 mAbs with known binding to RBD or N-terminal domain (NTD) tested for ADE, 131 mAbs bind exclusively to RBD, 154 mAbs bind to both RBD and NTD, and 44 mAbs bind solely to NTD (49) (https://covicdb.lji.org). Interestingly, about half of antibodies binding to RBD alone or to both RBD and NTD induced high ADE, showing no significant difference between these 2 groups. However, all antibodies specific to NTD alone did not promote ADE in contrast to antibodies specific to RBD (*P* < 0.0001, Fisher’s exact test). Importantly, all these NTD binders did not block binding to ACE2.

We next determined the contribution of individual mAb properties including binding to FcγR2a assessed by flow cytometry, blocking of RBD binding to ACE2, neutralization potencies (IC_50_), and antibody-dependent monocyte phagocytoses (ADMP) in ADE. The properties of mAbs were taken from the CoVIC database (https://covicdb.lji.org). Principal component analysis revealed clusters of mAbs with strong, moderate, and low ADE (Figure 3, E and F). Interestingly, ADE was strongly associated with the capability of mAbs to block RBD binding to ACE2 receptors. As expected, ADMP was strongly associated with binding to FcγR2a, and no correlation was observed between neutralization and binding to FcγR2a or ADMP. Furthermore, we found moderate negative correlation between neutralization and blocking of binding to ACE2 (Figure 3G).

### SARS-CoV-2 antigenic drift does not increase the overall ability of the antibodies to cause ADE.

**Figure 4:**
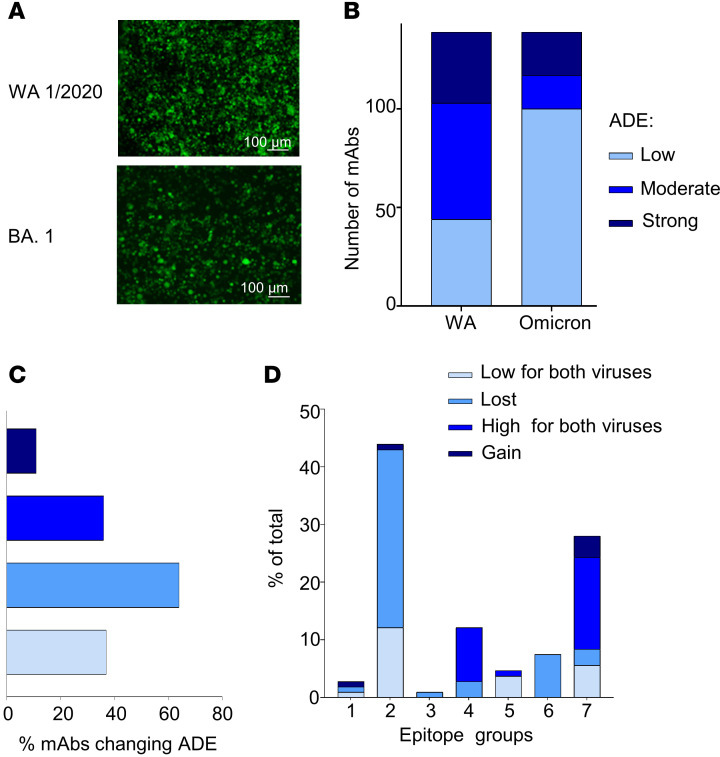
Antigenic drift of SARS-CoV-2 does not lead to increased ADE. (**A**) ADE in THP-1 cells caused by CoVIC-58 at 1 μg/mL infected with the original USA-WA1/2020 strain and the BA.1 (Omicron) strain. Scale bars: 100 μm. (**B**) Proportion of mAbs mediating low, moderate, and strong ADE for the USA-WA1/2020 and the BA.1 strain. (**C**) Percentages of mAbs that changed the extent of ADE in the presence of BA.1 compared with USA-WA1/2020. (**D**) Percentages of mAbs within epitope groups changing or keeping unchanged the degree of ADE for BA.1 compared with the USA-WA1/2020 strain.

To investigate the effect of SARS-CoV-2 antigenic drift on the viral ability to cause ADE, 139 mAbs from the CoVIC panel were tested for ADE with the recombinant USA-WA1/2020, in which the spike protein was replaced with the counterpart from the Omicron BA.1 strain, which is hereinafter referred as the BA.1 strain (Figure 4A). The proportions of mAbs that cause high and moderate ADE with the original USA-WA1/2020 strain were strongly reduced with the BA.1 strain, with a significant number of mAbs (64 in total) that no longer facilitated ADE. The fractions of mAbs that exhibited either low or moderate ADE were similar, at 74% for USA-WA1/2020 and 84% for BA.1 (Figure 4B), with a decreasing number of mAbs showing moderate ADE for BA.1. Only 8% of the tested mAbs increased their ability to mediate ADE with BA.1 compared with the original USA-WA1/2020 strain (Figure 4C). The changes in the ability to promote ADE with the BA.1 variant were observed within all epitope groups. We noted that among the mAbs that lost the ability to mediate ADE, the members with the biggest changes belonged to epitope groups 2 and 6 (Figure 4D). The greatest proportion of mAbs that maintained strong ADE or gained ADE belonged to epitope group 7. Overall, these data suggest that the tested BA.1 strain of SARS-CoV-2 does not have an increased ability to cause ADE with the CoVIC mAbs. Moreover, 64% of mAbs completely lost their ability to mediate ADE when tested with the BA.1 strain. The observed reduction of ADE could be due to the lesser antigenic match between the virus and the mAbs. It is possible that mAbs collected from patients infected with Omicron would demonstrate an ADE profile with Omicron comparable to what we observed for mAbs with the original USA-WA1/2020 strain.

### ADE is observed in multiple types of human immune cells.

**Figure 5:**
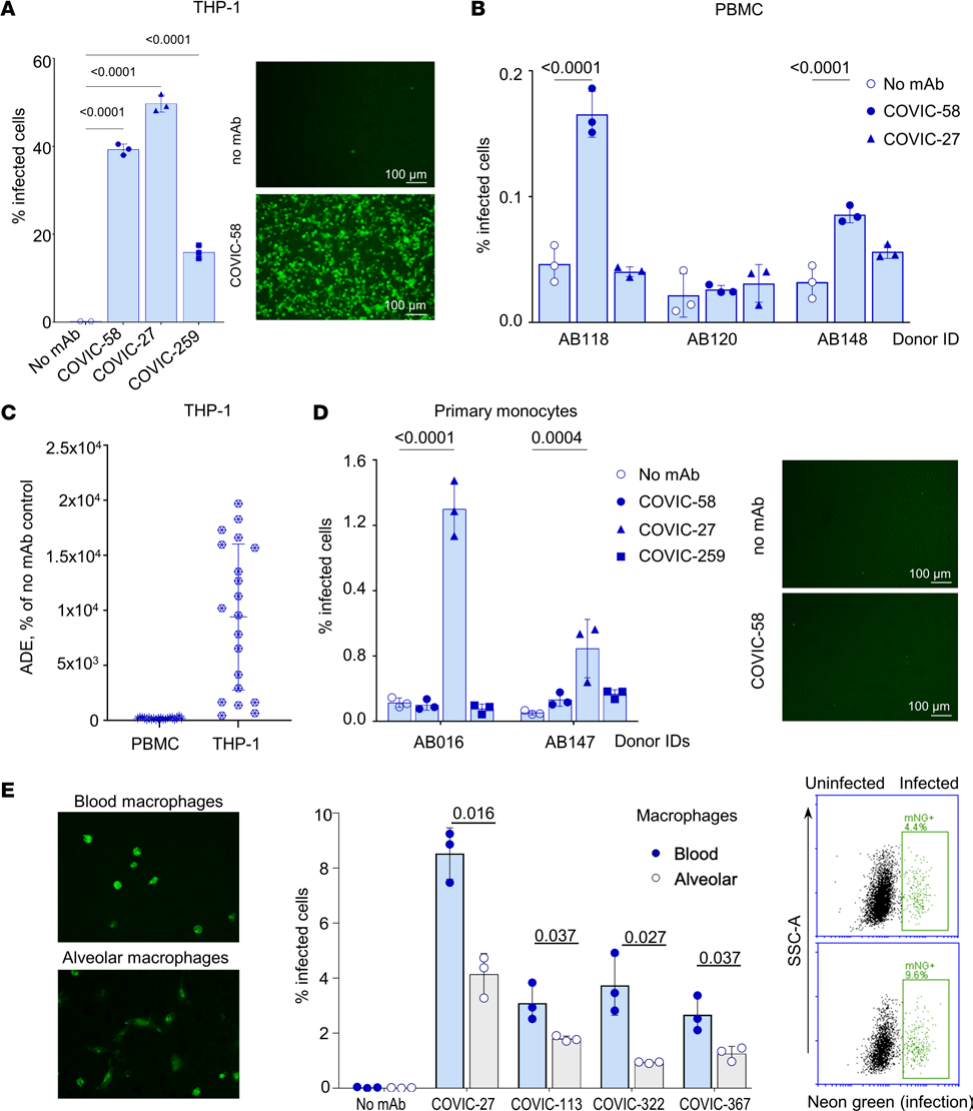
ADE observed in various types of immune cells. (**A**) THP-1, percentage infected cells, UV microscopy. (**B**) Human PBMC: percentage infected cells. (**C**) THP-1 monocytic cells, a panel of 20 mAbs, percentage no mAb control. (**D**) Primary human monocytes, percentage infected cells, UV microscopy. (**E**) Blood (top) and alveolar (bottom) types of monocyte derived macrophages from 1 donor treated with CoVIC-27: UV microscopy, percentage infected cells, primary flow cytometry data. Scale bars: 100 μm.

We demonstrated pronounced ADE in THP-1 cells, which served as model myeloid immune cells in our experiments. Our previous data demonstrated abundant SARS-CoV-2–specific RNA reads in myeloid but not lymphoid cells within the lungs of infected mice (57), consistent with similar findings in human studies (58, 59). For comparison of PBMCs and various myeloid immune cells, we selected a small cohort of mAbs with the notable capacity to enhance the infection (Supplemental Table 1) and tested them with THP-1, primary human PBMCs, primary human monocytes, and monocyte-derived macrophages (Figure 5). Interestingly, PBMCs were less susceptible to infection by SARS-CoV-2 than THP-1 (0.03% and 0.2% infected cells in the absence of mAbs, respectively) (Figure 5, A and B) and demonstrated a somewhat lower ability to promote ADE in the presence of mAbs (Figure 5C). The increases in the percentages of infected PBMCs never exceeded 4-fold, even with the mAbs inducing a 20-fold increase in the number of infected THP-1 cells. Primary human monocytes also demonstrated low susceptibility to SARS-CoV-2 (Figure 5D). To determine the susceptibility of blood and alveolar macrophages for ADE, we stimulated primary human monocytes for 6 days with macrophage CSF (M-CSF) or granulocyte-macrophage CSF (GM-CSF), respectively (60, 61). We observed a higher infectious rate in the presence of selected mAbs for both macrophage types, compared with other tested immune cells (Figure 5E). The ADE was more pronounced with blood macrophages, with up to 9.2% infected cells, compared with 0.03% in no-antibody control cells.

### ADE enhances spread of infection to non–immune cells.

**Figure 6:**
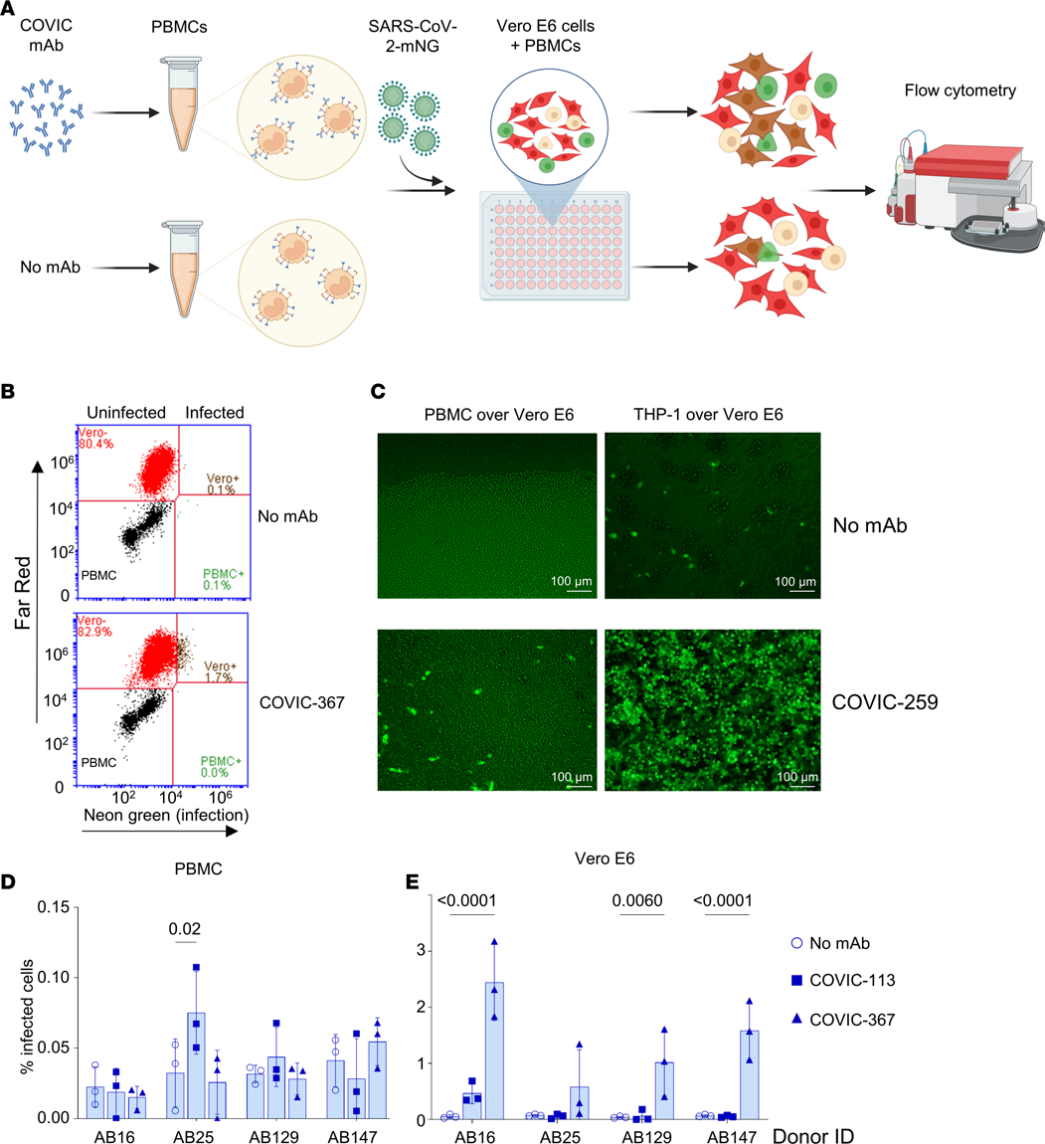
ADE enhances productive infection leading to viral dissemination. PBMCs of 4 donors were infected with mNG-SARS-CoV-2 in the presence of mAbs for 24 hours, washed 3 times, cocultured over the CellTrace far red dye–stained Vero E6 monolayer for the next 24 hours, collected, and analyzed by flow cytometry. (**A**) Schematic of the experiments. (**B**) Representative primary flow cytometry data. Green: infected PBMCs (mNG+); red: uninfected Vero E6 cells; brown: infected Vero E6 cells. (**C**) THP-1 is more susceptible to SARS-CoV-2 infection than PBMCs and can transfer virus more efficiently. Fluorescent microscopy. Scale bars: 100 μm. (**D** and **E**) Percentages of infected PBMCs (**D**) and Vero E6 cells (**E**) in PBMCs. Vero E6 cocultures quantified by flow cytometry: antibodies facilitate infection of Vero E6 cells.

Next, we were interested to determine whether ADE can contribute to the spread of the virus from immune cells to non–immune cells. To test this possibility, we prepared donor immune cells and acceptor immune cells as in our previous study with Ebola virus (10). To prepare donor cells, we infected THP-1 or PBMCs with SARS-CoV-2-mNG at MOI of 1 PFU per cell, applied the antibodies, incubated cells for 24 hours, and washed the cells 3 times with PBS to remove any unbound antibodies. To prepare acceptor cells, we stained Vero E6 monolayers with Far Red CellTrace. Next, the donor PBMCs were placed atop the acceptor Vero E6 cells, and the mixtures were incubated for 24 hours. To determine the percentages of infected acceptor cells, the monolayers and PBMCs were collected and analyzed by flow cytometry. The cells double-positive for far red and neon green were considered as infected Vero E6 cells (Figure 6, A and B). We found that the percentages of infected Vero E6 cells were significantly increased if PBMCs were preincubated with mAbs that cause ADE. For example, preincubation of PBMCs with CoVIC-367 resulted in an increase in the percentages of infected Vero-E6 cells from the background level of 0.04% (no mAbs during preincubation) to 2.44% (61-fold increase) (Figure 6, C–E). In a separate experiment, THP-1 cells were infected with SARS-CoV-2 at MOI of 1 PFU per cell in the presence of CoVIC-27, the Ebola virus–specific IgG VIC16, or without a mAb, incubated for 24 hours, washed 3 times, and cocultured with Vero E6 cells for 24 hours. Supernatants were purified from the cells by low-speed centrifugation, and the virus was quantified by plaque titration. The data demonstrated that CoVIC-27 increased viral titers in the supernatants 11.1-fold (Supplemental Figure 7). These data suggest that ADE caused by antibodies can facilitate spread of the virus throughout the body not only through Fc-bearing immune cells, but also through enhancement of infection of non–immune cells.

## Discussion

Here, we demonstrated that ADE mediated by SARS-CoV-2 spike protein–specific antibodies have characteristics in common with ADE observed for other viruses (62, 63), which is dependence on FcRs and on mAb concentration. While the magnitude of ADE was dependent on mAb concentration, the range of concentrations that cause ADE ranged between 0.1 and 10 μg/mL, and for most of the antibodies, the greatest enhancement was observed for the concentration of 1 μg/mL (Figure 1). We note that the SARS-CoV-2 infectivity of immune cells was very low to moderate, depending on a cell type, and the extent of ADE also varied widely, depending on a specific mAb and cell type. For Ebola virus antibodies, ADE was shown to be induced by strong neutralizers at sub-neutralizing concentrations (10). However, for SARS-CoV-2, the relation between the mAb concentration and the extent of ADE was not that obvious, as potent neutralizing antibodies could cause ADE even at neutralizing concentrations. Experiments with immune sera from patients with SARS-CoV demonstrated Fc receptor-dependent ADE in a human B cell line (63) and a human promonocytic cell line HL-CZ (14), where ADE was also dose dependent and was accompanied by increased apoptosis.

To explore the role of Fc domains in ADE, we selected a non-neutralizing mAb CR3022. In experiments in vitro, we observed a low ADE for the IgG1 variant, which was increased when the SDIE modification of the Fc domain was used. The mutation is known to enhance antibody-dependent cellular cytotoxicity (ADCC) and antibody-dependent cellular phagocytosis (ADCP) and provides higher binding to human FcγRs compared with WT. In contrast, the mutation N297D, which abrogates FcγR binding, abrogated ADE (Figure 2C), which is consistent with the data showing that blocking of FcγRI or FcγRIIa prevents ADE in THP-1 cells (54). FcγRs play a crucial role in antibody-mediated antiviral immunity. Development of SARS-CoV-2–specific Fc-engineered mAbs with limited disease-enhancing effects could enhance their clinical efficacy (11, 14, 64–66).

As our earlier published research showed, a nonmodified mAb CR3022 does not confer protection against SARS-CoV-2 in hamsters (54). Moreover, administration of the functionally enhanced Fc variant SDIE resulted in an increased pathology, enhanced viral replication in vivo, and weight loss, highlighting the pathological functions of the Fc-enhancing mutation (54). Interestingly, another modification of CR3022, G236A/A330L/I332E (GAALIE), did not result in disease-enhancing effects upon administration but failed to protect FcγR-humanized mice from lethal SARS-CoV-2 challenge, similar to the nonmodified mAb (67). We do not know whether the GAALIE modification can mediate ADE in vitro, but the SDIE modification enhanced infection in vitro and in vivo.

Among the mAbs with low ability to block viral binding to ACE2, the proportion of those causing ADE was low (Figure 2). In contrast, among the mAbs with a strong ability to block ACE2 binding, the percentage of those that induced ADE was significantly greater. The role of ACE2 binding in induction of ADE was further confirmed when ACE2 receptors were blocked either with polyclonal anti-ACE2 antibodies or ceftazidime, which reduced ADE. ACE2-independent ADE has been described for SARS-CoV (63) and may be explained by the lower affinity of SARS-CoV spike to ACE2 as compared with SARS-CoV-2 spike (68). Interestingly, increased shedding of ACE2 correlates with the severity of COVID-19 (68). Wang et al. (46) demonstrated that both FcγR and ACE2 are important for ADE in the case of SARS-CoV-2. ACE2 is involved in internalization of the virus after binding of RBD (69, 70), followed by endocytosis involving the cysteine protease cathepsin L (24, 71, 72). In our experiments, the lysosomal cathepsin inhibitor E64d significantly decreased ADE, but the addition of ACE2 blocking immune sera or a chemical ACE2 inhibitor led to a further reduction of ADE in the presence of enhancing antibodies, suggesting independent mechanisms of action of these inhibitors and their ligands.

We hypothesize that antibodies that directly interfere with binding of spike to RBD can promote ADE. The hypothesis is based on 3 sets of data: (a) ceftazidime, which sterically shields interaction of RBD with ACE2, prevented ADE (Figure 2D); (b) mAbs with low ability to block binding of RBD to ACE2 induced no or low ADE (Figure 2A); (c) all mAbs that demonstrated binding only to NTD do not promote ADE. The latter statement contradicts the “conformational” mechanisms of ADE assuming the involvement of NTD (73, 74), which could be not universal. The multivariable analysis revealed a strong association of ADE with the ability of mAbs to bind ACE2 (Figure 3E). ADE was less dependent on IC_50_ and binding to FcγR; of note, the latter parameter was closely linked to ADMP. The lesser dependence of ADE on virus neutralization IC_50_ distinguishes SARS-CoV-2 from other viruses: dengue (3, 4), respiratory syncytial virus (5), influenza (6), and Ebola (10).

**Figure 7:**
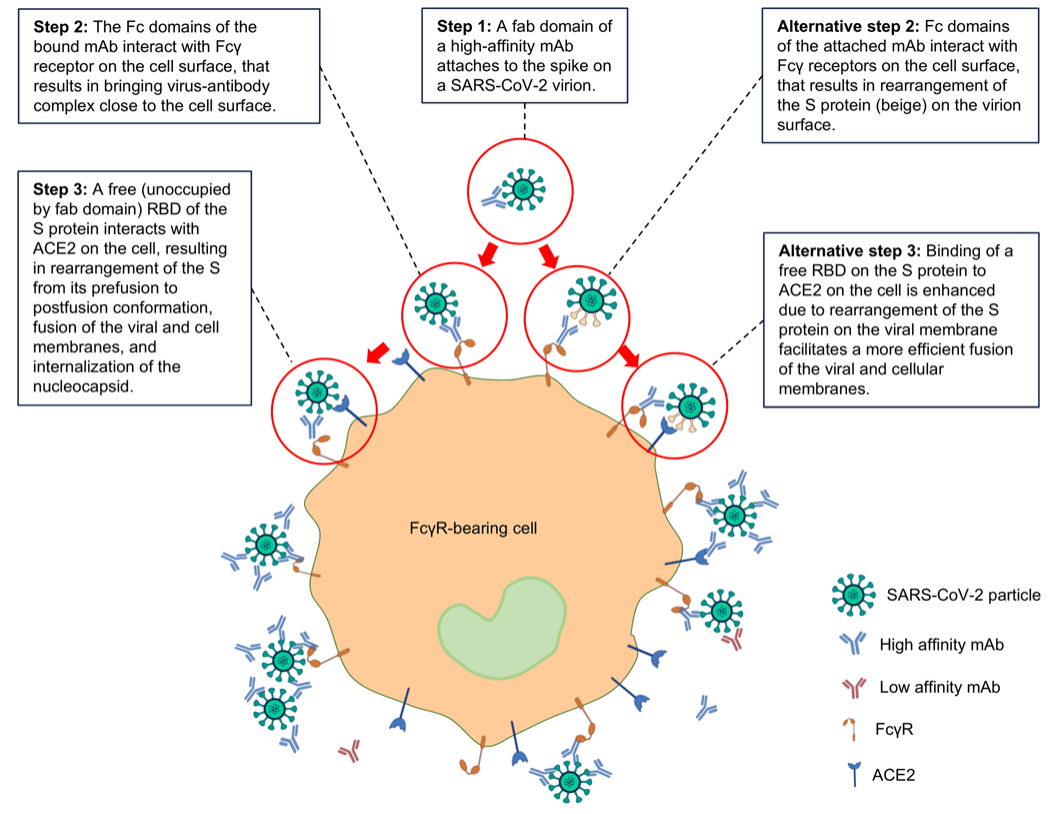
Graphic model. A model of mechanisms of ADE of SARS-CoV-2 infection.

Two mechanisms that are not mutually exclusive may explain the observed ADE mediated by high-affinity antibodies binding to the SARS-CoV-2 S protein (Figure 7). First, a high-affinity mAb binds viral RBD, resulting in a stable virus-antibody complex. The Fc domain of the bound mAb interacts with FcγR on the cell surface. This brings the virus-antibody complex into a close proximity to the cell membrane, enhancing viral attachment through interaction of an unoccupied RBD with ACE2. This step is FcγR dependent and can be blocked by FcγR inhibitors or by antibodies specific to FcγR. Binding of low-affinity mAbs could be insufficiently strong to initiate this process. This model is also consistent with the lack of ADE at high antibody concentrations, due to a complete neutralization of viral infectivity. It is also consistent with the ability of ceftazidime (35) and polyclonal antibodies against ACE2 to prevent ADE (Figure 2, C, E, and F). A second possible mechanism is that a strong binding of a mAb to RBD results in some rearrangement of unoccupied spike molecules on the viral surface, making them more accessible for antibodies that subsequently leads to increased engagement of Fc domains of immune cells. At high concentrations, antibodies occupy more RBDs, resulting in virus neutralization and lack of ADE. Furthermore, these models are consistent with the lack of ADE caused by NTD-specific mAbs, as NTD does not directly engage ACE-2.

The SARS-CoV-2 Omicron BA.1 variant emerged in 2021 and has multiple mutations in its spike protein, which led to a higher affinity for ACE2 and higher transmission compared with other SARS-CoV-2 variants (75, 76). The absence of ADE due to preexisting immunity to other coronaviruses does not alleviate concerns about possible ADE caused by administration of therapeutic mAbs isolated from patients infected with the original strain. To address this concern, we evaluated 139 antibodies with the BA.1 Omicron strain, and fortunately, half of the mAbs lost the ability to induce ADE in addition to 26% of the mAbs that did not promote ADE with either the original strain or BA.1 (Figure 4). Only 8% of the tested mAbs gained the ADE functions. Most of the mAbs that kept the ability to promote moderate and strong ADE belonged to the epitope group 7a and blocked RBD binding to ACE2 (1). Moreover, some antibodies with increased affinity to BA.1 spike demonstrated higher ADE with this strain. We should also take into account that the Omicron strain may be more dependent on the endocytic pathway to enter cells (77). We conclude that future mutations in SARS-CoV-2 are unlikely to increase ADE. Nonetheless, bispecific antibodies that target distinct epitopes (group 7) might exhibit diminished neutralization capacity for 1 epitope, potentially leading to ADE, and thus, their application should be approached with caution.

SARS-CoV-2 targets epithelial cells and poorly infects monocytes and macrophages in the peripheral blood (78) as well as the THP-1 monocytic cell line, even though THP1 cells carry both FcγR and ACE2 (14). Infection increases significantly in the presence of anti-spike mAbs or plasma from patients with COVID-19 (79), and peripheral monocytes in patients with COVID-19 start to differentiate into macrophages (78). Infection rates for most types of primary human immune cells in our experiments were low and only slightly increased in the presence of mAbs (Figure 5). However, macrophages were susceptible to SARS-CoV-2 and demonstrated a significant ADE.

Infection of macrophages was described previously for SARS-CoV (80, 81). Lung macrophages in patients with severe COVID-19 promote tissue infiltration of inflammatory monocytes, enhancing local inflammation, and may act as trojan horses propagating SARS-CoV-2 infection and spreading hyperinflammation across the lung (82). We demonstrated transfer of the infection from infected myeloid cells or PBMCs to uninfected Vero E6 cells, suggesting that the infection of immune cells is productive. Importantly, transfer of the infection was greatly enhanced by ADE-causing mAbs (Figure 6 and Supplemental Figure 7). These data contrast with previous observations that SARS-CoV-2 abortively infects monocytes and monocyte-derived macrophages (79, 83).

We previously demonstrated that enhancement of the Fc-mediated functions of a non-neutralizing SARS-CoV-2 antibody CR3022 resulted in ADE in vitro and worsened disease outcomes in animal studies (54). These findings underscore the critical role of Fc receptor engagement in modulating immune responses to SARS-CoV-2. The limitation of this study is the lack of in vivo data addressing the role of ACE2 interactions in ADE. In vivo systems encompass complex physiological processes, including immune cell trafficking and the expression of diverse viral entry receptors, which complicate the dissection of ADE mechanisms compared with controlled in vitro conditions. Furthermore, interspecies differences, such as variations in ACE2 binding affinity, may limit the direct translation of findings to humans. For example, transgenic mice may exhibit attenuated disease phenotypes, and the hamster is a better but not ideal model for severe human COVID-19 due to species-specific differences in viral susceptibility and immune regulation. Additionally, polyclonal antibody responses may show greater ADE in vivo, potentially leading to compartmentalized increases in viral load within specific organs or tissues. Future in vivo studies should carefully consider these variables to better understand the mechanisms and implications of ADE.

In conclusion, we demonstrate that even though infection of myeloid cells with SARS-CoV-2 is low, it may contribute to virus dissemination by infection of non–immune cells. Moreover, certain antibodies may strongly enhance spread of the virus by myeloid cells. These effects can contribute to inflammatory reactions and disease severity. Antibodies of different epitope specificities can promote ADE, and their ability to enhance infection is less related to their neutralization potencies, unlike other viruses, and more dependent on an efficient prevention of binding of spike to ACE2. Thus, ADE caused by SARS-CoV-2 relies not only on the interaction of antibodies with FcγR but also on their ability to block binding of spike to ACE2, resulting from two cooperating effects.

## Methods

### Sex as a biological variable.

Our studies used deidentified human PBMCs with unknown sex. Sex was not considered as a biological variable, as we are unaware of publications showing the effect of sex on the repertoire of SARS-CoV-2–specific antibodies or susceptibility of immune cells to SARS-CoV-2.

### Cell lines.

Vero-E6 cells (derived from epithelial cells of a green monkey kidney) were obtained from American Type Culture Collection (ATCC) (CRL-1586). Cells were maintained in MEM supplemented with 10% FBS and 0.1% gentamycin sulfate (Corning) at 37°C, 5% CO_2_. THP-1 cells (human monocytic leukemia cell line) were obtained from ATCC and cultured in RPMI 1640 (Thermo Fisher Scientific) supplemented with 10% FBS (HyClone) at 37°C with 5% CO_2_. Primary human immune cells were derived from blood. Blood samples were collected from 8 anonymous healthy adult donors at the UTMB blood bank. Buffy coats from blood samples were used for isolation of PBMCs by density gradient centrifugation in Ficoll (Histopaque; Sigma-Aldrich). CD14+ monocytes were purified using anti-human CD14 antibody-labeled magnetic beads and magnetic LS columns (Miltenyi Biotec) and used immediately or further differentiated into macrophages. Isolated monocytes typically showed greater than 90% positivity for CD14 staining by flow cytometry. Next, 10^7^ monocytes in 10 mL of medium were cultured in 75 cm^2^ flasks. IMDM (Gibco) was supplemented with 10% FBS (HyClone) and either 5 ng/mL of human GM-CSF (PeproTech) or 50 ng/mL of M-CSF (PeproTech) to differentiate into blood-circulation or alveolar type macrophages, respectively. The cells were cultured for 6 days until there were visible changes in morphology. The full medium complemented with GM-CSF or M-CSF was replaced every 48 to 72 hours.

### Viruses.

The recombinant SARS-Co-V-2-mNG was constructed on the genetic background of an infectious cDNA clone derived from clinical strain WA1 (2019-nCoV/USA_WA1/2020, GenBank ID: MN985325; GISAID: EPI_ISL_404895) containing an mNG reporter gene as previously described (47). The Omicron spike mutations (BA.1 variant, GISAID EPI_ISL_6640916) were engineered using a PCR-based mutagenesis protocol (84). Both viruses were provided by Pei-Yong Shi (UTMB). All work with SARS-CoV-2 was performed in the BSL-3 facility of the Galveston National Laboratory.

### Neutralization assay.

CoVIC mAbs at concentrations 200–0.048 μg/mL (7-point concentration curve) were preincubated with SARS-CoV-2-mNG (passage number not to exceed 3) for 1 hour in 96-well plates, and the antibody-virus mixtures were applied to Vero-E6 cell monolayers at MOI of 0.005 PFU per cell. At 48 hours after infection, mNG-positive (infected) cells were quantified using a BioTek Cytation 7 plate reader, with excitation wavelength 485 nm and emission wavelength 528 nm. The curves of the relative infectivity versus the serum dilutions (log_10_ values) were plotted using GraphPad Prism 9. A nonlinear regression method with log (inhibitor) versus response-variable slope (4 parameters) model with bottom and top parameters constrained to 0 and 100, respectively, was used to determine the dilution fold that neutralized 50% of mNG SARS-CoV-2 (defined as IC_50_) in GraphPad Prism 9.

### ADMP assay.

Vero E6 cells serving as target cells were infected with SARS-CoV-2-mNG1 at MOI of 0.1 PFU per cell and incubated at 37°C in 5% CO_2_ for 48 hours under BSL-3 containment. Then, the virus-infected Vero E6 cells were harvested with trypsin-EDTA (0.25%) and preincubated at 37°C with 10-fold serially diluted mAbs for 90 minutes. Monocytes isolated from the buffy coat layer of PBMCs using CD14 microbeads were used as effector cells and were added to the Vero E6 cell-antibody mixtures at a 4:1 effector/target ratio. The cells were then cocultured at 37°C in 5% CO_2_ for 4 hours. After coculture, the cells were stained with CD14-PE (clone 63D3, BioLegend) and CD66b-APC (clone G10F5, BioLegend) for 10 minutes at room temperature. Cells were washed with PBS and fixed with 4% paraformaldehyde twice as per the BSL-3 laboratory protocol. After fixation, the cells were analyzed in an LSR Fortessa with FlowJo software version 10.8 (Tree Star). The percentage phagocytosis of virus-infected cells by monocytes was calculated from flow cytometry data with monocytes being identified as CD14+ CD66b– SSC-A^int^. Phagocytic scores were determined using the formula: % cells with mNG signal × geometric MFI / 10,000. The fold-change in percentage phagocytosis or phagocytic score of any mAb relative to the no-mAb control was used to categorize ADMP as follows: less than 1.8 fold-change, weak; 1.8 to 3.0 fold-change, moderate; greater than 3 fold-change, strong.

### ADE mediated by mAbs.

THP-1 cells (ATCC) and mNG USA-WA1/2020 or Omicron-spike SARS-CoV-2 mAbs were diluted to the final concentrations of 10, 1, and 0.1 μg/μL and added to the bottom of 96-well flat-bottom plates. Next, 100,000 THP-1 cells were seeded per each well, and the plates transferred into the BSL-3 containment. Viral suspensions containing 300,000 PFU of SARS-CoV-2-mNG were added into the wells, resulting in the MOI of 3 PFU per cell. The total medium volume in each well was 200 μL. Cells were then cultured for 24 hours at 37°C in a CO_2_ incubator. After incubation, the cell suspensions were collected into FACS tubes. Cells were fixed in 4% paraformaldehyde for 24 hours, followed by a second fixation in fresh paraformaldehyde, and taken out of the BSL-3 containment according to the approved standard operation procedure. Then, fixed cells were centrifuged, resuspended in PBS, and analyzed by analytical flow cytometry using a BD Biosciences Accuri C6 Plus Personal Flow Cytometer. For each antibody at each concentration, ADE was calculated using the formula [% NG-positive cells in presence of antibody / % NG-positive cells in virus control (no antibodies)] * 100%. ADE was categorized relative to virus control for 1 μg/μL mAbs as strong, greater than 100-fold; moderate, 10–100-fold; low or none, less than 10-fold to no-mAb control, which corresponds to greater than 20% infected cells, 2%–20% infected cells, less than 2% infected cells, respectively; in the absence of mAb, approximately 0.18% of cells were infected. Primary human immune cells (PBMCs, monocytes, or macrophages) were treated the same way, but only one concentration of antibodies, 1 μg/μL, was tested.

### Inhibitors for ADE blocking.

The following reagents were used: polyclonal antibody against ACE2 (503602 LEAF purified anti-human ACE2 antibody clone Poly5036), demonstrated by the manufacturer to block binding of SARS-CoV-2 spike protein to ACE-2, mAb specific for ACE2: CL4035, AMAB91262 (Sigma-Aldrich), mAb specific for CD64: 217620-100UG clone 10.1 (MilliporeSigma), mAb specific for CD16: SAB4700274 3G8 (MilliporeSigma), human mAb specific for FcγRII: MABF925 clone AT10 (Sigma-Aldrich), ceftazidime (MedChemExpress, HY-B0593), and E64D protein (R&D Systems, 4545/1).

### Flow cytometry analysis of mAb binding to FcγRs.

A multiplex assay was used to determine FcR binding as described by Ateyo et al. (54, 85). A 2-step carbodiimide reaction was used to couple antigen to Magplex Luminex beads. Beads were activated for 30 minutes at room temperature using 100 mM monobasic sodium phosphate, pH 6.2, with 5 mg/mL N-hydroxysulfosuccinimide and 5 mg/mL ethyl dimethylaminopropyl carbodiimide hydrochloride. Beads were then washed with 50 mM 2-(N-morpholino) ethanesulfonic acid (MES), pH 5.0, and incubated with 25 μg of antigen in 50 mM MES, pH 5.0, for 2 hours on a rotator. The coupled beads were blocked in blocking buffer (PBS, 0.1% BSA, 0.02% Tween-20, 0.05% azide, pH 7.4). After blocking, coupled beads were washed in PBS-Tween, resuspended in PBS, and stored at 4°C. For the detection of FcR binding, FcRs with an AviTag were biotinylated using a BirA500 kit (Avidity) per the manufacturer’s instructions. Coupled beads were diluted to a concentration of 100 microspheres per antigen/μL in 0.1% BSA in PBS. Antibodies were serially diluted in 0.1% BSA in PBS; mixed with diluted beads in a black, clear-bottom, 384-well plate; and incubated at 4°C for 16 hours, shaking at 160*g*. After the incubation, plates were washed with 0.1% BSA in PBS. FcRs were incubated with streptavidin-PE (Prozyme, PJ31S) for 10 minutes. PE-labeled FcRs were added to plates and incubated for 1 hour at room temperature on a shaker. Plates were washed with 0.1% BSA in PBS and resuspended in Qsol Buffer (Intellicyt). Fluorescence was acquired on the Intellicyt iQue. The antibody binding to FcγR was evaluated using Luminex, and AUC was calculated for the MFI values.

PBMCs were infected with SARS-CoV-2-mNG at MOI of 1 PFU per cell for 24 hours, washed with FACS buffer (2% BSA in PBS), stained with Zombie Aqua (BioLegend), washed, and stained for the following anti-human surface markers: CD3 (APC/Fire750, BioLegend) CD19 (PerCP/Cy5.5, BioLegend, clone H1B19), and CD56 (Brilliant Violet 605, BioLegend, clone 5.1H1). The surface staining was performed at 4°C for 45 minutes in the presence of FACS buffer. Cells were washed with FACS buffer and analyzed using a BD Biosciences FACSymphony A5 SE instrument. Data were analyzed using FlowJo (v10).

### Biolayer interferometry analysis of blocking of RBD binding to ACE2 by antibodies.

The ability of antibodies to block the interaction between RBD and ACE2 was assessed by biolayer interferometery (BLI) using a FortéBio Octet HTX instrument (Sartorius). The detailed methods have been described previously (1, 49). Briefly, SARS-CoV-2 RBD as well as HSA (Abcam) as a negative control for parallel reference subtraction were immobilized through amine coupling onto Amine Reactive 2nd Generation (AR2G) biosensors (Sartorius) to a loading density not to exceed Δλ = 0.7 nm by using a set threshold option. Then, antibody binding at 20 μg/mL and ACE2 binding at 27.5 μg/mL to RBD-loaded biosensors were sequentially tested in triplicate. Additionally, the binding of ACE2 to immobilized RBD with no prebound antibody was measured. Data analysis was performed using Data Analysis HT 12.0 (CFR11) software (Sartorius). The parallel reference (HSA) subtracted average response of ACE2 binding to immobilized RBD without prebound antibody was set as 0% blocking. The percentage blocking was calculated as percentage decrease in ACE2 binding due to antibodies prebound to RBD compared with RBD binding to ACE2 with no bound antibodies. Data were quality controlled for reporting by applying a preset data acceptance criterion. For this, the percentage coefficient of variation (%CV) of triplicate measurements of percentage ACE2 blocking needed to be below 20% if the percentage ACE2 blocking was above an empirically determined lower limit of detection of 13%. This was not applied if the percentage of ACE2 blocking was below the lower limit of detection.

High-throughput surface plasmon resonance epitope binning was reported previously (1). A classical sandwich assay format was used to determine epitope groups using a Carterra LSA HT-surface plasmon resonance instrument equipped with a CMDP or HC30M sensor chip. Assays were carried out at 25°C in HBSTE-BSA running buffer (10 mM HEPES pH 7.4, 150 mM NaCl, 3 mM EDTA, 0.05% Tween-20, supplemented with 0.5 mg/mL BSA). Samples were deposited on the sensor chip using 2 microfluidic modules, a 96-channel print-head (96PH), and a single flow cell (SFC). The chip surface was prepared with 25 mM MES pH 5.5 with 0.05% Tween-20 as a running buffer and activated with a freshly prepared solution of 130 mM 1-ethyl-3-(3-dimethylaminopropyl) carbodiimide plus 33 mM N-hydroxysulfosuccinimide in 0.1 M MES pH 5.5 using the SFC. Antibodies (10 μg/mL diluted with 10 mM sodium acetate, pH 4.25) were immobilized using the 96PH for 10 minutes, followed by quenching of unreactive esters with a 7-minute injection of 1 M ethanolamine-HCl (pH 8.5) using the SFC. The array was used for the binning analysis with the HBSTE-BSA buffer as the running buffer and sample diluent. In each cycle, a 4-minute injection of RBD antigen (1.8 μg/mL; 50 nM; aa 318-591 based on GenBank sequence MN908047) was immediately followed by a 4-minute injection of the analyte antibody at 30 μg/mL (200 nM for IgG constructs). After each cycle, the surface was regenerated with double pulses (17 sec/pulse) of 10 mM glycine (pH 2.0). Epitope software supplied with the LSA instrument was used to process and analyze the data. Briefly, unprinted locations on the array were used to reference the data, and each binding cycle was normalized to the RBD capture level. Analyte antibody binding levels just after the end of the injection were compared with that of a buffer-alone injection. Significant increases in signals compared with buffer controls were designated as sandwiches and correspond to non-blocking activity. Heatmaps depicting blocking relationships of analyte/ligand pairs were used to visualize competition results. Clones having similar competition patterns clustered together in a dendrogram that was used to assign shared communities. In competition maps, light and dark teal indicate non-blocking and blocking, respectively, and black shading indicates self.

### Statistics.

Statistical analyses and generation of graphs were performed using GraphPad Prism version 9. One-way ANOVA with multiple comparisons (Tukey’s test) with post hoc analysis (Kolmogorov-Smirnov test normality distribution), principal component analysis, Spearman’s correlation matrix of variables, and Dunnett’s multiple-comparison test were used for statistical data analysis. Fisher’s exact test was used to compare the ability of NTD- and RBD-specific antibodies to promote ADE.

### Study approval.

Blood samples from deidentified healthy donors were collected at the UTMB blood bank and used according to a clinical protocol approved by the UTMB IRB (#10-221).

### Data availability.

Quantitative data are available in the Supporting Data Values file. All other data are available upon request.

## Author contributions

NAK, EOS, and AB were responsible for conceptualization. NAK and AB wrote the original draft. AB was responsible for project administration and study supervision. EOS and AB acquired funding. NAK, EOS, AB, SP, KK, KH, CA, SMD, KL, DB, SLS, GDT, HA, and GA contributed to the methodology, formal analysis, and review and editing of the manuscript.

## Funding support

This work is the result of NIH funding and is subject to the NIH Public Access Policy. Through acceptance of this federal funding, the NIH has been given a right to make the work publicly available in PubMed Central.

- National Institute of Allergy and Infectious Diseases, NIH U19 AI142790-02S1 (to EOS and AB).
- UTMB intramural funds (to AB).

## Supplementary Material

Supplemental data

Supporting data values

## Version 1 02/23/2026

Electronic publication
