# Supplementary material for: SARS-CoV-2 antibody–dependent enhancement of infection depends on antibody binding to both ACE2 and Fc receptors
Source: JCI Insight. 2026 Feb 23;11(4):e197773. doi: 10.1172/jci.insight.197773 (PMC12956010; doi:10.1172/jci.insight.197773)
Supplement: Supplemental data [file jciinsight-11-197773-s171.pdf]

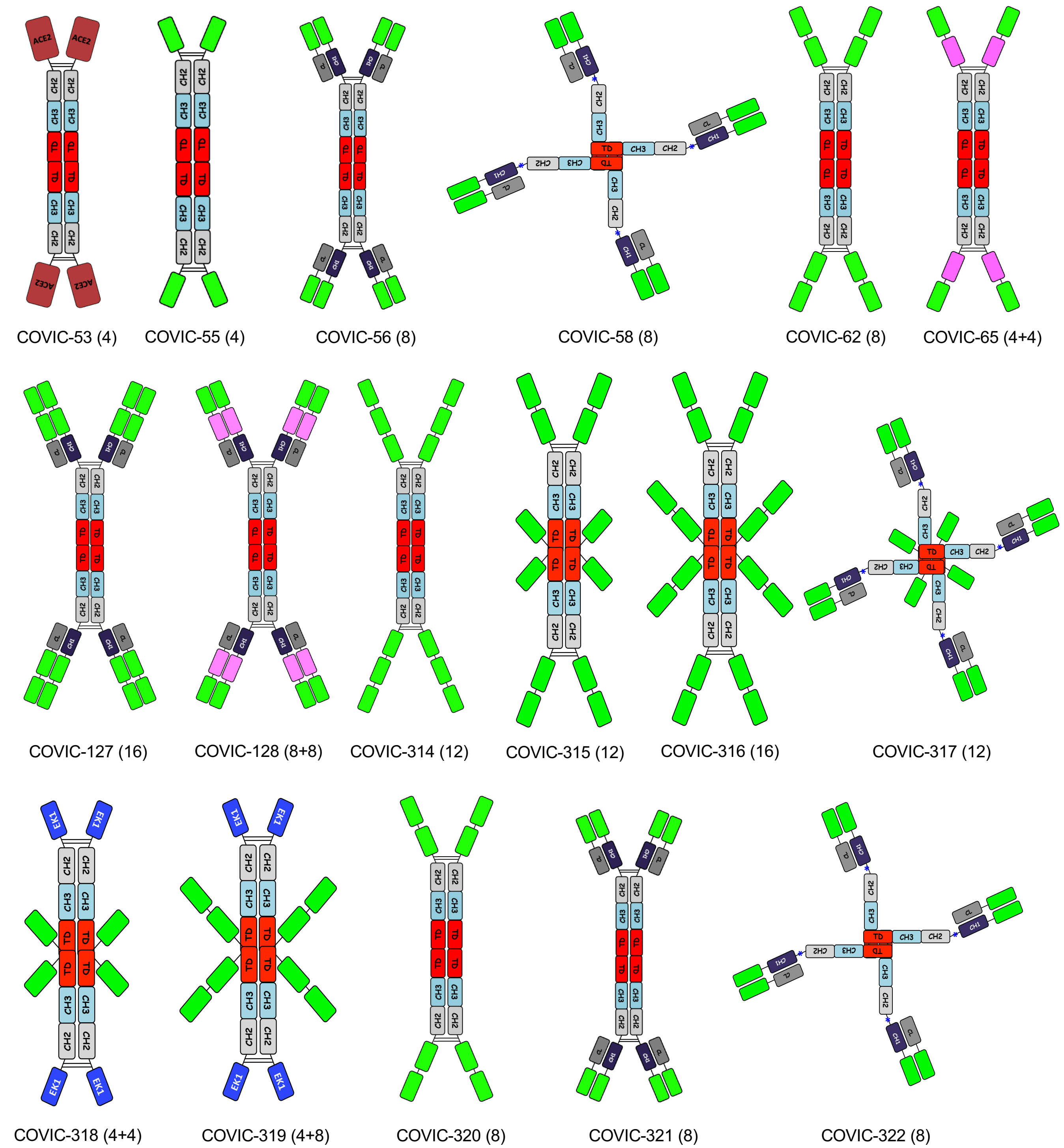

Suppl. Fig. 1. Schematic representation of multivalent antibodies specific to SARS-CoV-2 S protein. The molecules, which differ in size, geometry, flexibility, and binding domain valency, were generated using a self-assembling tetramerization domain (red) fused to the C-terminus of the immunoglobulin Fc domain (light blue and gray) (1). COVIC-53 utilizes the human ACE2 extracellular domain as a decoy receptor to bind the SARS-CoV-2 receptor-binding domain (RBD, dark brown). All other COVIC molecules employ an anti-SARS-CoV-2 VHH binding domain (green), which targets the RBD of SARS-CoV-2. COVIC-65 and COVIC-128 are multivalent bispecific molecules that contain VHH binding domains for both SARS-CoV-2 and MERS-CoV (pink) domain. The VHH SARS-CoV-2 binding domains in COVIC molecules 314-322 incorporate a humanized version of the VHH binding domain. Additionally, COVIC-318 and COVIC-319 contain the EK1 peptide binding domain, shown in blue, which functions as a membrane fusion inhibitor (2). For each antibody, the binding domain valencies is indicated in parentheses. More detailed information about the antibodies can be provided upon request.

References

1. Miller, A., et al.. Multimeric antibodies with increased valency surpassing functional affinity and potency thresholds using novel formats. *mAbs*, 2020. 12(1):1752529.

2. Yu, D., et al. Pan-coronavirus fusion inhibitors possess potent inhibitory activity against HIV-1, HIV-2, and simian immunodeficiency virus. *Emerg. Microbes Infect*., 2021 (1), p. 810-821.

CoVIC ID	FcγR2a	FcγR3a	FcγR2b	FcγR3b
CoVIC 53	5.45	5.44	4.8	4.96
CoVIC 55	6.37	6.28	6.01	6.42
CoVIC 56	6.33	6.23	5.96	6.34
CoVIC 58	5.29	5.52	3.75	3.65
CoVIC 62	6.32	6.24	5.97	6.36
CoVIC 65	6.32	6.27	5.97	6.42
CoVIC 127	5.96	5.95	5.57	5.96
CoVIC 128	6.17	6.1	5.71	6.11
CoVIC 314	5.91	6.01	4.68	6.14
CoVIC 315	5.76	5.94	4.15	6.03
CoVIC 316	5.74	5.91	4.11	6.02
CoVIC 317	4.72	5.68	4.09	3.95
CoVIC 318	5.69	5.95	4.11	6.32
CoVIC 319	5.85	5.92	4.16	6.28
CoVIC 320	5.99	6	4.9	6.32
CoVIC 321	5.39	5.84	4.14	4.55
CoVIC 322	5.54	5.89	4.18	4.53
Mean of modified mAbs	**5.81**	**5.95**	**4.84**	**5.67**
Mean of dataset represented in Fig. 1C	**5.88**	**5.80**	**5.55**	**5.66**

Suppl. Fig. 2. Binding of the modified antibodies to Fc-receptors. Binding affinity for the indicated Fc receptors was measured by flow cytometry. MFI values for areas under the curve (AUC) were calculated based on serial antibody dilutions.

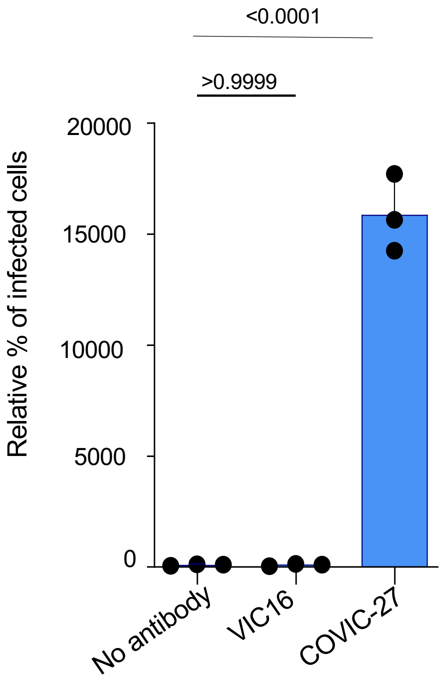

Suppl. Fig. 3. Treatment of SARS-CoV-2-infected THP-1 cells with an irrelevant antibody VIC-16 resulted in no ADE similarly to cells which received no antibody.

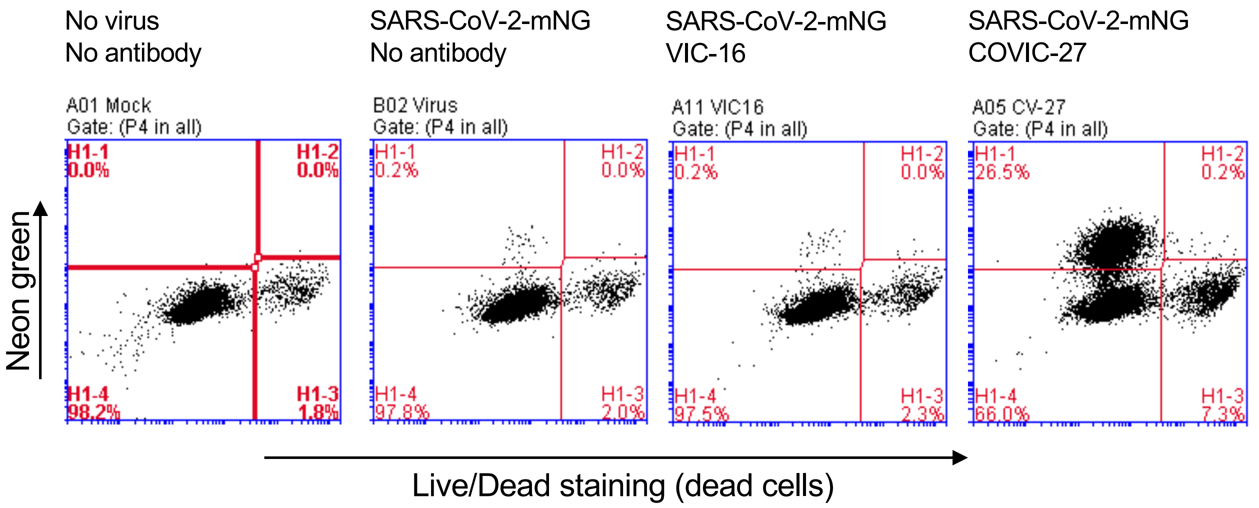

Suppl. Fig. 4. Viability of THP-1 cells treated with antibodies and infected with SARS-CoV-2-mNG. THP-1 cells were treated with PBS, isotype control antibody specific for Ebola virus (VIC16) or COVIC-27, infected with SARS-CoV-2 mNG and incubated for 24 h. Cells were stained with FarRed Live/Dead dye, washed, fixed and analyzed by flow cytometry. Quadrants: H1-1 – infected live cells, H1-2 – infected dead cells, H1-3 – uninfected dead cells, H1-4 – uninfected live cells.

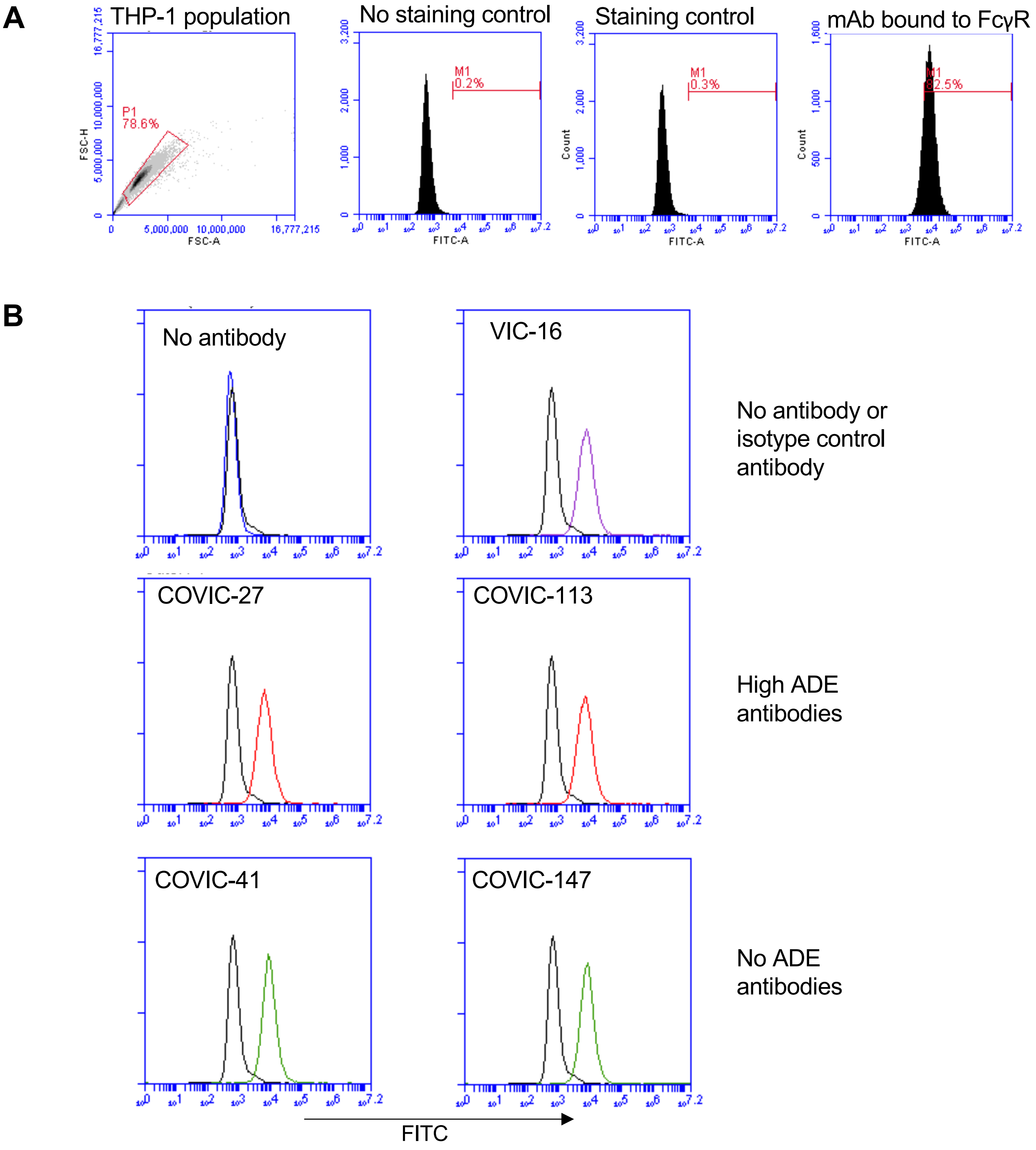

Suppl. Fig. 5. Binding of mAbs to Fcγ receptors on THP-1 cells determined by flow cytometry using FITC-labeled anti-human IgG1 as a secondary antibody. A. Gating strategy. **B.** Top row: no antibodies (black peak) or an irrelevant IgG1 specific for Ebola virus VIC-16 (purple peak). Middle row: SARS-CoV-2-specific antibodies which cause high ADE (red peaks). Bottom row: SARS-CoV-2-specific antibodies which do not cause ADE (green peaks). Unstained cells are shown as black peaks. These data show that antibodies which cause ADE and antibodies which do not cause ADE bind Fcγ receptors with comparable efficiency.

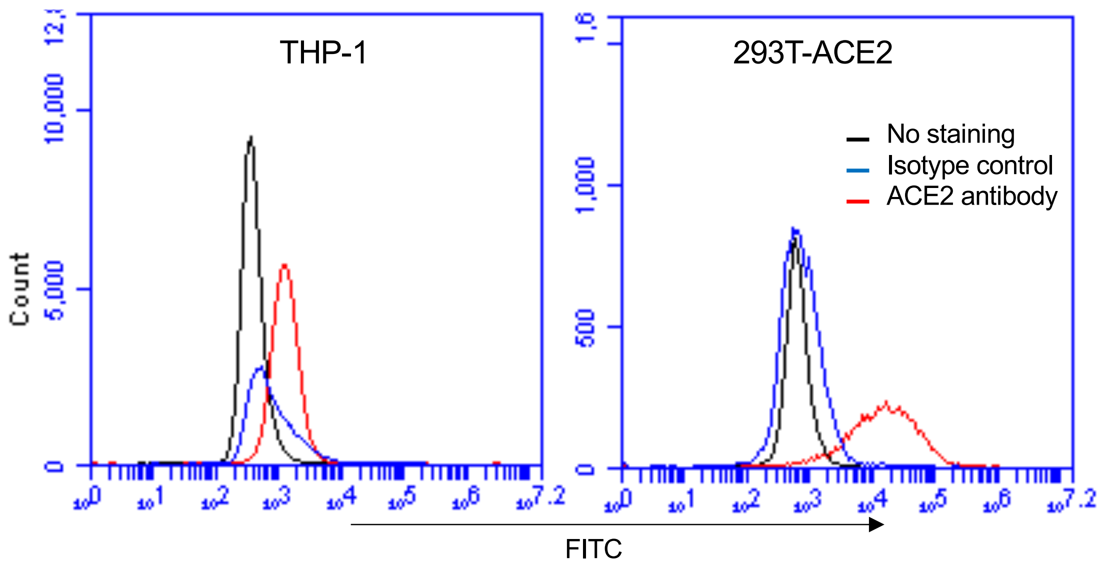

Suppl. Fig. 6. THP-1 cells used in ADE assays express ACE2. A. THP-1 cells were stained with FITC-labeled mAb specific for ACE2 [ACE2 (E11): sc-390851 FITC, Santa Cruz biotechnology]. **B.** 293T cell line engineered to express ACE2, which was used as a positive control. An antibody to normal mouse IgG conjugated to FITC (normal mouse IgG1-FITC; Santa Cruz Biotechnology, sc-2855) was used isotype control.

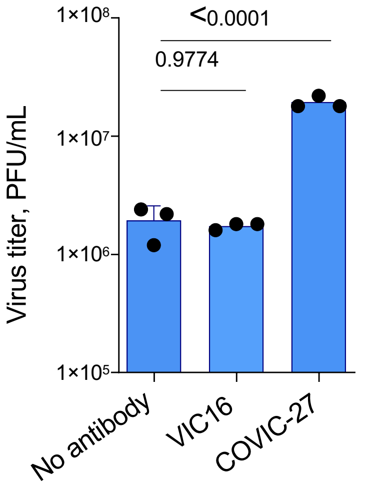

Suppl. Fig. 7. **Viral titers in supernatants of Vero E6 cells after 24 h-long co-culture with infected PBMCs.** Limit of detection 20 PFU/ml.

Supplemental Table 1. Properties of the selected panel of CoVIC mAbs.

CoVIC ID	Isotype	Epitope Commu-nity	Sub-Epitope Commu-nity	IC50, ng/mL	Binding to FcγR2aR, MFI*	Binding to FcγR3a, MFI	Binding to FcγR2b, MFI	Binding to FcγR3b, MFI	Blocking of binding of RBD to ACE2, %	ADE, %**
CoVIC-27	IgG1	3	3a	20700	6.19	6.13	5.87	6.24	88.6	339
CoVIC-58	IgG1	7	7a	87.4	5.29	5.52	3.75	3.65	100	237
CoVIC-113	IgG1	7	7c	25000	6.06	6.11	5.48	6.03	27.2	107
CoVIC-259	IgG1	1	1	57.4	6.31	6.32	5.91	6.19	100	160
CoVIC-322	IgG1	7	7a	71.1	5.54	5.89	4.18	4.53	100	81
CoVIC-367	IgG1	4	4c	346	nt	nt	nt	nt	45.3	197

* MFI values were calculated based on serial antibody dilutions and expressed as areas under the curve (AUC).

** ADE values were determined in THP-1 cells at 1 μg/ml of mAb and expressed as fold increase of the number of infected cells vs no antibody control,

nt - not tested
